# Impact of Carcinogenic Chromium on the Cellular Response to Proteotoxic Stress

**DOI:** 10.3390/ijms20194901

**Published:** 2019-10-03

**Authors:** Leonardo M. R. Ferreira, Teresa Cunha-Oliveira, Margarida C. Sobral, Patrícia L. Abreu, Maria Carmen Alpoim, Ana M. Urbano

**Affiliations:** 1 Department of Surgery and Diabetes Center and Sean N. Parker Autoimmune Research Laboratory, University of California, San Francisco, San Francisco, CA 94143, USA; leonardo.ferreira@ucsf.edu; 2 CNC-Center for Neuroscience and Cell Biology, University of Coimbra, UC-Biotech, Biocant Park, 3060-197 Cantanhede, Portugal; teresa.oliveira@uc-biotech.pt; 3 Department of Life Sciences, University of Coimbra, 3000-456 Coimbra, Portugal; margaridasobral2014@hotmail.com; 4 Instituto de Medicina Molecular João Lobo Antunes, Faculty of Medicine, University of Lisbon, 1649-028 Lisbon, Portugal; patricia.abreu@medicina.ulisboa.pt; 5 Department of Life Sciences, Center of Investigation in Environment, Genetics and Oncobiology (CIMAGO) and CNC-Center for Neuroscience and Cell Biology, University of Coimbra, 3000-456 Coimbra, Portugal; mcalpoim@gmail.com; 6 Department of Life Sciences, Molecular Physical Chemistry Research Unit and Center of Investigation in Environment, Genetics and Oncobiology (CIMAGO), University of Coimbra, 3000-456 Coimbra, Portugal

**Keywords:** carcinogenesis, hexavalent chromium, heat shock proteins, HSP70, HSP90, HSP inhibitor, occupational lung carcinogen, proteotoxic stress, stress response, unfolded protein response

## Abstract

Worldwide, several million workers are employed in the various chromium (Cr) industries. These workers may suffer from a variety of adverse health effects produced by dusts, mists and fumes containing Cr in the hexavalent oxidation state, Cr(VI). Of major importance, occupational exposure to Cr(VI) compounds has been firmly associated with the development of lung cancer. Counterintuitively, Cr(VI) is mostly unreactive towards most biomolecules, including nucleic acids. However, its intracellular reduction produces several species that react extensively with biomolecules. The diversity and chemical versatility of these species add great complexity to the study of the molecular mechanisms underlying Cr(VI) toxicity and carcinogenicity. As a consequence, these mechanisms are still poorly understood, in spite of intensive research efforts. Here, we discuss the impact of Cr(VI) on the stress response—an intricate cellular system against proteotoxic stress which is increasingly viewed as playing a critical role in carcinogenesis. This discussion is preceded by information regarding applications, chemical properties and adverse health effects of Cr(VI). A summary of our current understanding of cancer initiation, promotion and progression is also provided, followed by a brief description of the stress response and its links to cancer and by an overview of potential molecular mechanisms of Cr(VI) carcinogenicity.

## 1. Hexavalent Chromium: Applications, Chemical Properties and Biological Implications

Chromium (Cr), a transition metal, is the 21st most abundant chemical element in Earth’s crust. It can exist in a variety of oxidation states, from −2 to +6, but most of these states are too unstable to exist in any significant amount [1]. In nature, Cr exists mostly in the trivalent oxidation state, Cr(III), but it can also be found in the hexavalent oxidation state, Cr(VI). Cr(VI) compounds have a wide range of applications and are extensively used as pigments for textile dyes, paints, inks and plastics, corrosion inhibitors, leather tanning agents and wood preservatives, amongst other uses [2,3]. Due to the low natural abundance of Cr(VI) compounds, all Cr(VI) used in industrial and commercial applications must be produced from Cr(III) found in chromite ores.

Cr(III) compounds are essentially innocuous and are widely used as nutritional supplements [4,5], although their beneficial health effects have been questioned by the European Food Safety Authority [6]. On the contrary, exposure to Cr(VI) compounds is associated with numerous adverse health effects, mostly to the skin and respiratory system. Importantly, the International Agency for Research on Cancer (IARC), the National Toxicology Program (NTP) and other highly respected regulatory agencies have classified Cr(VI) compounds as lung carcinogens [7,8,9].

The highest human exposures to Cr(VI) occur in the chemical, metallurgical and refractive chrome industries, through dermal contact and inhalation of dusts, mists and/or fumes. In addition, significant exposure can occur during welding, casting and cutting of stainless steel and other chromium-containing metals and alloys, as Cr(VI) can be given off as a by-product [8]. The general population and the wildlife, particularly those living in the vicinity of chromate industries, may also be exposed through inhalation of ambient air or ingestion of contaminated drinking water. Leaching of wastewater from industrial waste disposal sites and landfills may also contaminate drinking water. In addition, Cr(VI) compounds are continuously released to ambient air as exhaust emission products in fuel combustion and cigarette smoke. Milling and demolition are additional sources of environmental contamination, as Cr(VI) compounds are present, as impurities, in Portland cement [3].

The different toxicities of Cr(III) and Cr(VI) compounds can be rationalized in terms of their physico-chemical properties. Namely, their ability to cross biological membranes and, ultimately, induce intracellular damage is determined by their sizes, structures and charges. At physiological pH, Cr(VI) exists mostly as chromate anions (CrO_4_^2−^). Being isostructural with the sulfate and phosphate anions, the chromate anions released from Cr(VI) compounds move easily across cellular membranes using the anion transport system [10,11]. By contrast, the larger size and octahedral structure of the Cr(III) ions prevent them from using this transport system. Still, a very small fraction of insoluble Cr(III) salts are taken up by the cells, mostly by phagocytosis [12]. Poorly water-soluble chromates with a particle size of less than 5 μm can also be phagocytosed and will gradually dissolve in the intracellular milieu [8].

Postmortem microscopic analysis of lung tissue and biopsy samples from chromate industry workers revealed that particulate Cr(VI) compounds tend to deposit at the bronchial bifurcations [13,14,15]. Postmortem studies further showed that tumors tend to develop centrally in the lung, with the most frequent histological type of Cr(VI)-induced lung cancer being squamous cell carcinoma [16]. Thus, it has been argued that Cr(VI) compounds are particularly dangerous when inhaled in the form of particulates, as their slow and constant dissolution ensures a long lasting exposure of lung epithelial cells to chromates. However, as an excess risk of lung cancer was observed among workers exposed to Cr(VI) compounds of diverse solubilities, it is likely that all Cr(VI) compounds are endowed with a similar carcinogenic potential [8].

Suspicions of a link between Cr exposure and lung cancer were first raised in the late nineteenth century, when an increased incidence of this type of cancer was observed among Scottish chrome pigment workers [17]. Since then, evidence in favor of this link has steadily accumulated. Yet, it was only in the 1980s that Cr, more specifically Cr(VI), was firmly established as a human lung carcinogen. This classification triggered an intense search for the cellular and molecular mechanisms underlying Cr(VI)-induced lung cancer. Naturally, the lines of research followed over time have been influenced by contemporary theories of carcinogenesis. For contextualization, our current understanding of cancer initiation, promotion and progression will be briefly discussed in the next section, with an emphasis on the roles played by different stresses.

## 2. Cancer Initiation, Promotion and Progression: The Critical Importance of Oxidative, Proteotoxic and Genotoxic Stresses

According to the current paradigm, carcinogenesis is driven by stepwise genetic mutations and concomitantly enhanced and uncontrolled cell proliferation. DNA damage is believed to be in the genesis of this process by creating a transformed cell, which over the course of additional genomic and cellular insults becomes a fully malignant and metastatic cancer cell [18]. In spite of the creation of elaborate genetic mouse cancer models [19] and large-scale sequencing efforts for many cancer types [20], the process of carcinogenesis remains, for the most part, poorly understood.

**Figure 1:**
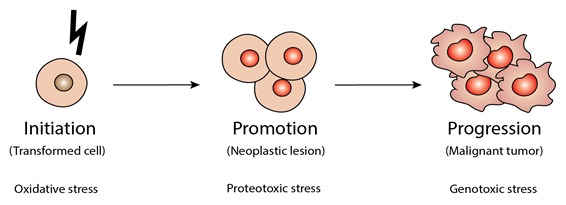
The different types of stress associated with the three stages of carcinogenesis. Carcinogenesis has been traditionally divided in three stages: initiation, promotion and progression. Different types of cellular stress have been implicated in these stages. Oxidative stress and reactive oxygen species (ROS) damage proteins and membranes, and induce DNA mutations. Incipient cancer cells at the promotion stage harbor an increasing number of DNA mutations, resulting in dramatically higher levels of mutant proteins that induce proteotoxic stress. Transition to a fully malignant phenotype, i.e., progression, is thought to require chromosomal instability and resulting karyotypic abnormalities, inducing genotoxic stress. Of note, all types of stress indicated (oxidative, proteotoxic and genotoxic) play roles in all three stages of carcinogenesis described above; their relative importance likely differs amongst different types of cancer.

Traditionally, carcinogenesis has been divided into three phases: initiation, promotion and progression [21] (Figure 1). Initiation entails the acquisition of mutations in proto-oncogenes and tumor suppressor genes. Significantly, incipient cancer cells feature a deranged metabolism, leading to high levels of reactive oxygen species (ROS) and, consequently, oxidative stress [22,23,24,25] (Figure 1). ROS damage not only DNA, but also proteins and membrane lipids. Yet, ROS also play a role in cellular signaling, promoting cell proliferation and adaptation to the hypoxic conditions often found in the tumor microenvironment [26]. In particular, mitochondrial ROS inactivate inhibitory phosphatases (e.g., PTEN), unleashing the PI3K/AKT cell survival and growth pathway, and prolyl hydroxylases (e.g., PHD2). In turn, this inactivation stabilizes hypoxia inducible factors (HIF), concomitantly triggering angiogenesis. Next, cancer cells enter a promotion phase, when mutations in oncoproteins such as growth factor receptors and kinases gradually lead to independence from extracellular growth factors [27]. As mutations often disrupt a protein’s ability to fold [28], accumulation of increasingly larger amounts of mutated proteins represents yet another type of cell intrinsic stress—proteotoxic stress [29] (Figure 1). This type of stress can be created by any structural alteration that may lead to protein misfolding and aggregation. Ultimately, incipient cancer cells form a solid tumor mass, creating with it a tumor microenvironment. Here, cancer cells reprogram stromal cells to produce tumorigenic cytokines, chemokines and tissue-remodeling metalloproteinases [30], inhibit anticancer immune responses [31] and recruit blood vessels via angiogenesis to sustain their continued growth [32]. The tumor microenvironment also creates a host of cell extrinsic stressors, including hypoxia, acidosis and nutrient deprivation [33,34,35,36]. Malignant tumors are also characterized by rampant chromosomal instability and aneuploidy, caused by chromosome segregation errors during mitosis. Such extensive damage leads to genotoxic stress. While genotoxic stress leads to p53-induced apoptosis in normal cells, in malignant cells it is tolerated and subverted, giving rise to a mosaic of genomic mutations and karyotypic abnormalities in solid tumors [37,38,39].

## 3. Links between the Cellular Response to Stress and Carcinogenesis

Carcinogenesis entails the acquisition of a growing ability to survive in the face of cellular stress levels that normal cells are unable to withstand. There is now a growing perception that this ability results, at least in part, from a subversion of the cellular systems that evolved to protect normal cells against stress. This section will briefly describe one of these systems, the so-called stress response, a homeostatic system to combat proteotoxic stress that is found across all three domains of life [40]. It also includes a discussion of the links between the stress response and cancer.

### 3.1. Note on Nomenclature

Several of the studies discussed in this review were carried out at a time when very little was known regarding heat shock proteins (HSP) and their role in the cellular response to stress. Back then, HSP were named based on their approximate subunit molecular weights, as determined by polyacrylamide gel electrophoresis. For instance, the designations Hsp90 and HSP90 were used interchangeably to describe any protein with an approximate subunit molecular weight of 90 kDa whose expression was rapidly and strongly induced by stress. Since then, the number of known stress-responsive proteins, some of which constitutively expressed, has expanded enormously. Many of the now known isoforms share identical subunit molecular weights and it is often not possible to retrospectively identify the specific isoform(s) being described in the earlier studies. In this review, we use the abbreviation Hsp when referring to a clearly identified isoform (e.g., Hsp72), whilst HSP abbreviates either one or more unspecified isoforms of a given family or the family as a whole (e.g., HSP90 will be used to describe an unidentified isoform of an approximate subunit molecular weight of 90 kDa or the HSP90 family as a whole).

To complicate matters further, as some stress-responsive proteins were not initially classified as HSP; they were given unrelated names. Currently, up to ten different names can be found in the literature for the same gene product [41]. Aiming at reducing inconsistencies and increase clarity, Kampinga and collaborators put forward, already in 2009, new guidelines for the nomenclature of the human HSP [41]. Unfortunately, this nomenclature has not yet been widely adopted, remaining unfamiliar to most readers. In Table 1, which summarizes all studies covered in this review on the impact of Cr(VI) on components of the stress response, genes and gene products are presented as found in the corresponding papers. Nonetheless, whenever an unambiguous identification was possible, the new nomenclature was added, between brackets, following the name used in the original paper.

### 3.2. The Stress Response: Basic Concepts

The cytoprotective effects of the stress response are mediated by the heat shock proteins (HSP). These molecular chaperones promote proper protein folding, translocation and degradation, as well as the assembly and disassembly of protein complexes [57,58]. In mammals, heat shock factor 1 (HSF1) is the main transcriptional regulator of the stress response [59,60].

In eukaryotic cells, the stress response comprises different sub-systems, which fulfil organelle-specific functions, such as the unfolded protein response (UPR), which operates in the endoplasmic reticulum (ER) [61], and the mitochondrial unfolded protein response (UPRmt). The ER is a major site for the synthesis, folding, modification and transport of secretory and transmembrane proteins, as well as for the assembly of protein complexes [62,63]. Incorrect protein maturation can occur even under physiological conditions, due to, among other causes, the very high protein concentrations normally found in the ER (~100 mg/mL [64,65]). ER stress, i.e., the incapacity of this organelle to manage its load of client proteins, is further aggravated under conditions of nutrient deprivation, hypoxia, augmented ROS levels and acidic extracellular milieu, amongst others [66]. Of note in the context of the present review, these conditions are often found in the tumor microenvironment. Furthermore, certain cancers, such as the B cell-derived malignancy multiple myeloma, produce extremely high levels of immunoglobulins, which translates into protein overload and consequent ER stress [67].

Accumulation of unfolded or misfolded proteins triggers the UPR, which signals transient attenuation of protein translation, while increasing the ER capacity of protein folding and degradation of misfolded proteins [64,65,68]. Amongst the molecular chaperones involved in the re-establishment of protein homeostasis (i.e., proteostasis) are numerous glucose-regulated proteins (induced by glucose starvation), including Grp78, which is the most abundant ER-resident chaperone, and Grp94 [64,65,68,69,70]. Grp78 and Grp94 are the ER homologues of, respectively, HSP70 and HSP90 proteins. After a certain time, proteins that remain aggregated, misfolded and/or unassembled are targeted for ER-associated degradation (ERAD), leading to their translocation from the ER to the cytosol to be degraded by the ubiquitin-proteasome machinery [71]. If ER stress becomes chronic, abnormal calcium signaling from ER to mitochondria and apoptotic pathways can be activated [72].

In eukaryotes, the metabolic energy required to sustain cellular processes, including stress-induced adaptations, is generated mostly in the mitochondria. Interestingly, mitochondria are closely connected to the ER through mitochondria-associated membranes (MAMs), which allow the exchange between these two organelles of lipids, calcium ions (Ca^2+^) and, possibly, ROS. It has also been suggested that MAMs are involved in glucose homeostasis [73]. ER and mitochondrial stress pathways seem to be interconnected, as a mitochondria resident HSP90, tumor necrosis factor receptor-associated protein 1 (TRAP1), has been associated with UPR in the ER [74,75]. Also, p53-upregulated PUMA and NOXA [76] and Lon protease [77], which is also a chaperone [78], seem to be part of a signaling pathway that transmits ER dysfunction to the mitochondria. ER stress, amino acid depletion, excessive ROS levels, oxidative phosphorylation (OXPHOS) perturbation, impaired complex assembly (mitonuclear protein imbalance) and the accumulation of misfolded proteins impair mitochondrial protein import efficiency and lead to nuclear translocation of the activating transcription factor associated with stress (ATF) and subsequent activation of the UPRmt [79,80,81]. In the nucleus, ATF mediates the transcription of genes involved in the re-establishment of mitochondrial function, mitochondrial proteostasis and protein import efficiency [82,83]. Resistance to ER and mitochondrial stresses can contribute to carcinogenesis [84,85].

### 3.3. Cancer and the Stress Response

It has been known for some time that most types of tumors display augmented HSP levels [86]. Increased HSF1 activity likely contributes to the augmented HSP levels, yet it has been reported that HSP gene promoters can also be activated by the oncogenic transcription factor c-MYC, as well as by loss of the tumor suppressor protein p53 [87]. Strikingly, deletion of HSF1 in mice bearing mutations in the *Ras* oncogene and *Tp53* tumor suppressor gene protected them from tumor formation [88].

Specific HSP have been directly implicated in p53 inactivation and malignant transformation [89], as well as in cancer invasiveness and resistance to chemotherapy [90]. For instance, HSP90 overexpression, which was observed in a broad spectrum of cancers, correlated with tumor growth, metastatic potential and resistance to chemotherapy [86,91,92]. This observation led to the proposal that tumors develop an “addiction” to HSP90 [93,94]. It is noteworthy that, unlike other HSP, HSP90 proteins are not necessary for the correct folding of newly synthesized proteins. Instead, their main role is to stabilize meta-stable proteins, ultimately suppressing the formation of protein aggregates. Importantly, numerous oncoproteins are HSP90 clients [95]. Chief among these are several receptor tyrosine kinases and steroid hormone receptors, such as the human epidermal growth factor 2 (HER2), associated with uncontrolled cellular proliferation [92,96], telomerase, an enzyme required for immortalization [97], AKT, involved in the deregulation of the apoptosis [98], hypoxia-inducible factor 1-alpha (HIF-1α), essential for angiogenesis [99] and the metabolic shift observed in tumors [22,92,100], and matrix metalloproteinases (MMPs), crucial for successful tissue invasion and metastasis [101]. According to the "HSP90 addiction hypothesis", cancer cells need an increased pool of HSP90. This increased pool is critical to retrieve essential proteins that became misfolded due to extensive proteotoxic stress and to allow increasingly more mutated oncoproteins and tumor suppressor proteins to function, by preventing their misfolding and degradation.

Remarkably, HSP90 proteins have also been found in the extracellular milieu, where they act as potent stimulators of immune responses [102]. Unsurprisingly, HSP90 is currently being explored as a target for cancer therapy. There are currently 73 clinical trials employing HSP90 inhibitors registered in ClinicalTrials.gov. Nevertheless, no HSP90 inhibitor has been approved for cancer treatment yet [103].

Altogether, the stress response emerges as a double-edged sword: evolved to protect cells from menaces to homeostasis, it might constitute, in its extreme, one of the main mechanisms behind cancer cells’ formidable resilience. Several questions remain open. How much cellular stress is required for it to have an impact on carcinogenesis? Do qualitatively different levels of stress play distinct roles in cancer? HSP activation is exquisitely sensitive to cellular stress-inducing agents. Studying the links between the stress response and carcinogenesis will answer these and other questions and contribute to a more detailed understanding of cancer.

## 4. The Molecular Mechanisms of Hexavalent Chromium Carcinogenicity: A Brief State of the Art 

Genetic and epigenetic mechanisms likely play a critical role Cr(VI) carcinogenesis. This view is supported by the observation of genetic lesions in both the lung cells of chromate workers and in cultured cells exposed to different Cr(VI) concentrations [11,13,15,16,104,105,106,107,108,109]. Thus, the initial observation, in test tube experiments, that Cr(VI) is mostly unreactive towards DNA (and most other biomolecules) puzzled researchers. However, it is now known that, following its rapid cellular uptake, Cr(VI) undergoes a multi-step reduction that generates a variety of species that react extensively with biomolecules, namely Cr(III), which is the final reduction species, and the unstable intermediates Cr(IV) and Cr(V) [110,111]. Under physiological conditions, ascorbate accounts for about 90% of Cr(VI) reduction, but non-protein thiols, such as glutathione and cysteine, also contribute significantly to its reduction [112]. Thus, Cr(VI) reduction generates additional reactive species, such as carbon-based radicals from ascorbate, and thiyl radicals from glutathione and cysteine. The generation of ROS [113,114] is still a matter of debate, as it has been argued that the methods employed for detection of Cr(VI)-induced ROS were not adequate and that the Cr(VI) concentrations employed in those studies were too high to be of biological relevance [115]. Among the Cr(III)-DNA complexes formed are Cr(III)-DNA adducts, DNA-protein crosslinks and DNA interstrand crosslinks [11,115].

Cr(VI) exposure can result in DNA damage by both direct and indirect mechanisms. For instance, Cr(VI) exposure may lead to loss of thiol redox control through interference with antioxidant defense systems [116]. This and additional lines of evidence, namely the observation of 8-hydroxy-2’-deoxyguanosine formation in rat lungs following intratracheal administration of Cr(VI) [108], suggest that Cr(VI) exposure can damage DNA through the generation of oxidative stress [50,117,118]. Additionally, altered ROS levels affect gene expression [119].

DNA damage can also result from a direct interaction of these biomolecules with Cr(III), generating different types of Cr(III)-DNA adducts. By restraining the normal DNA replication and transcription processes, these adducts activate the various cellular DNA repair systems in a lesion-dependent manner. Cr-DNA monoadducts are preferentially repaired by the base excision repair (BER) system in coordination with the apurinic/apyrimidinic (AP) site repair system [120]. The transient single-strand breaks (SSB) that are formed are then promptly repaired by the cooperative action of DNA polymerase β (Polβ) and the X-ray cross-complementing group 1 (XRCC1) complex [121]. Cr(III)-DNA-protein crosslinks and DNA inter/intrastrand crosslinks (ICLs) require recruitment of other DNA repair systems, namely the nucleotide excision repair (NER) system [122]. Mutations in key proteins involved in these DNA repair systems have been described both in Cr(VI)-induced lung cancer patients and in cultured cells exposed to Cr(VI) compounds, impairing their ability to remove chromium-DNA adducts [122]. In addition to SSB formation, double-strand break (DSB) induction by the mismatch repair (MMR) system may drive genomic instability, either as a direct result of the repair systems or due to delayed repair and concomitant cell cycle arrest which, in the case of Cr (VI), often uncouples karyokinesis from cytokinesis [3,123]. Hirose and co-workers reported a high incidence of microsatellite instability (MSI), a particular type of genomic instability that specifically affects the microsatellites, in lung cancers from chromate-exposed workers [3,124,125]. However, a similar finding could not be observed upon in vitro exposure of human lung epithelial cells to Cr (VI) [126].

Unsurprisingly, the impact of Cr (VI) on the signaling pathways that underlay cell proliferation, differentiation and death has been the focus of multiple research studies, but a clear picture is yet to emerge. While an in-depth discussion of these studies is beyond the scope of this review, it is noteworthy that the sequences targeted in Cr(III)-DNA and Cr(III)-histidine-DNA lesions in the *TP53* gene are identical, with both types of adducts formed at –NGG- sequences at mutational hotspots in lung cancer. These findings suggest that Cr(III)-DNA adduct formation contributes to the *TP53* mutations observed in lung carcinogenesis [127].

Cancer has been traditionally viewed as a genetic disease, but it is now increasingly clear that non-genetic events can also be critical players in carcinogenesis. For instance, Cr(VI)-induced lesions may contribute to the onset of inflammatory lung disease, which in turn predisposes to lung cancer, as illustrated by the strong correlation between lung cancer and both bronchitis and interstitial lung diseases [128,129]. In line with this hypothesis, it was reported that zinc chromate nanoparticles induce bronchiolar cell apoptosis and mucosal injury, later progressing to alveolar and interstitial pneumonitis. It was also found that inflammatory cytokines, such as IL-6 and TNF-α, and activation of the survival pathway AKT were involved [120,122,123,124,125,127,128,130,131]. Another study revealed that, in vitro, progression to higher malignant states in Cr(VI)-induced carcinogenesis is mediated by the inflammatory cytokines IL-6 and G-CSF and Activin A released by stromal cells, with the concomitant activation of STAT3 and WNT signaling pathways [132].

## 5. The Impact of Hexavalent Chromium on the Stress Response 

As mentioned previously, there is a growing perception that the stress response may be a critical player in carcinogenesis. Cr(VI) may promote proteotoxic stress and, ultimately, activate the stress response through various mechanisms. For instance, changes in protein conformation may result from their direct interaction with Cr(III). Conformational changes may also be a consequence of oxidative stress, as it may originate incorrect disulfide bonds and other forms of protein modification [116]. The induction of mutations, as found in in vivo and in vitro systems [133], can also compromise the correct folding of the affected proteins [28].

The number of published studies on the impact of Cr(VI) on the stress response is still small. In addition, most of these studies did not specifically address the role of the stress response on carcinogenesis. Namely, some of the earlier studies were exploiting the then relatively recent array cDNA technology to simultaneously investigate multiple gene pathways that might be affected by Cr(VI) exposure [48,56]. Table 1 summarizes all the studies covered in this review.

The first observation of an effect of Cr(VI) on the stress response was made in 1998, on a molecular toxicology study aimed at developing a sensitive biological system for the rapid detection of low levels of environmental pollutants [49]. Using a radiolabeled antisense RNA probe, the authors found that, at mildly cytotoxic concentrations, a 6 h exposure to Cr(VI) increased Hsp72 mRNA levels, in HepG2 and HT29 cells. These results confirmed that HSP activation is exquisitely sensitive to Cr(VI) exposure, as changes in Hsp72 transcript levels could be detected for Cr(VI) concentrations as low as 0.5 μM. Of note, mRNA levels were determined 3 h after the stressing exposure, as it was observed that, after heat shock, transcript levels strongly increased in the first 3 h, then decreasing to nearly basal levels 6 h after shock. In an independent study, protein levels peaked instead at 6 h after exposure [134], stressing the importance of conducting adequate time courses.

The second report of Cr(VI) impacting the stress response came from a study aimed at identifying metal-responsive promoters and, ultimately, new signal transduction pathways that might be modulated by exposure to this and other environmental pollutants [50]. To this end, 13 recombinant HepG2 cell lines, each of which stably transfected with a specific stress-responsive promoter regulating the expression of the chloramphenicol acetyl transferase (CAT) reporter gene, was exposed, for 48 h, to different Cr(VI) concentrations. Intracellular levels of CAT protein were determined immediately after exposure. In the case of the two cell lines that had been transfected with HSP promoters, specifically the HSP70 and Grp78 gene promoters, a subcytotoxic Cr(VI) concentration induced CAT upregulation, even though statistical significance was only reached in the cell line transfected with the HSP70 promoter. At a higher Cr(VI) concentration, CAT protein levels were further augmented, yet this was accompanied by a dramatic decrease in cell viability. The results of this study highlighted the different susceptibilities of these two HSP to Cr(VI).

Cr(VI) is a lung carcinogen and, as such, studies conducted on human epithelial lung cells should be particularly informative. In the A549 cell line, established from a human lung adenocarcinoma, a 2 h Cr(VI) exposure upregulated the transcript levels of TRAP1, the mitochondrial homologue of Hsp90 [56]. However, the Cr(VI) concentration used in this study was extremely high and would likely cause massive cell death for longer exposures. Therefore, the results of this study must be interpreted with caution. Nonetheless, it was recently reported, also in the A549 cell line, that a much lower Cr(VI) concentration upregulated Grp78 protein levels, again peaking at 6 h of Cr(VI) exposure [55]. The exquisite sensitivity of Grp78 to Cr(VI) is noteworthy. In L-02 hepatocytes, Grp78 mRNA levels were increased after a 24 h exposure to Cr(VI) in the low micromolar range [54]. In the same cell line, a similar exposure regimen, which was found to induce significant cytotoxicity, decreased the protein levels of both HSP70 and HSP90 [53].

Two studies have been conducted in the BEAS-2B cell line, established from normal human bronchial epithelium, which is the main target of Cr(VI) carcinogenicity. Both studies used Cr(VI) concentrations that did not cause overt cytotoxicity. The first study aimed at identifying specific and sensitive biomarkers of toxic metal exposure [46]. One significant finding was the extreme specificity of the Cr(VI) effects: of the 1200 gene transcripts analyzed, only 44 had their expression altered after a 4 h Cr(VI) exposure. Of the 44 genes affected, 3 encoded HSP (HSP40, HSP60 and HSP90A) and were all down-regulated. The transcript levels of all other HSP analyzed (HSP27, HSP-70, HSP70.1, HSP-71) remained unchanged, giving further support to the perception that the impact of Cr(VI) is isoform-specific.

The second study employing BEAS-2B cells investigated the impact of Cr(VI) on the expression of the Hsp72 and Hsp90α isoforms at both the transcript and protein levels [47]. Importantly, this study unveiled decoupling of mRNA and protein levels for both Hsp72 and Hsp90α. After a 48 h incubation with Cr(VI), Hsp72 mRNA levels were decreased, whereas Hsp72 protein levels remained unchanged. For Hsp90α, mRNA levels were unaltered, whereas protein levels were decreased. This decoupling is likely multifactorial, potentially involving critical post-transcriptional regulators, such as RNA binding proteins and microRNAs [135,136]. Protein stability and turnover may also have to be taken into account [137,138]. Thus, in future studies, it will be important to conduct detailed time-courses of the effects of Cr(VI) on gene expression at both levels.

There are another two cellular studies on the impact of Cr(VI) on HSP70 [51,52] and one on the impact of this carcinogen on HSP90 [52]. Altogether, these studies clearly show that this impact is dependent on both the cellular model employed and on the experimental design.

Another study, conducted in rat lung epithelial cells, showed the impact of Cr(VI) on additional HSP isoforms, namely Hsp10 and Hsp105, whose protein levels were increased after a 24 h incubation, which was shown to produce significant cytotoxicity [42]. Two other studies, one employing HaCaT [43] cells and the other employing human primary skin fibroblasts [44], unveiled Cr(VI) ability to alter the phosphorylation state of HSP27. Of note, aberrant phosphorylation of HSP27 has been associated with cancer [139]. In HaCaT cells, HSP27 expression was upregulated by Cr(VI) at both transcript and protein levels, but the phosphorylation of this HSP was decreased [43]. On the contrary, levels of phosphorylated HSP27 were found to be increased in Cr(VI)-exposed in human primary skin fibroblasts [44]. This apparent contradiction might be explained by differences in cell model, Cr(VI) concentration and/or time of exposure.

In the only two in vivo studies conducted to date, one employing ICR mice [45] and the other Sprague-Dawley rats [48], Cr(VI) administration induced HSP expression. In ICR mice, Cr(VI) intraperitoneal injection increased liver HSP27 and HSP70 protein levels. In Sprague-Dawley rats, Cr(VI) intratracheal instillation increased HSP70 mRNA levels in the lungs, whereas these levels were unaltered in the liver. HSP60, Grp75 and Grp94 mRNA levels, on the other hand, were unaffected in both lungs and liver. In fact, none of the 216 genes assessed had their liver mRNA levels altered, whereas changes in lung mRNA levels were observed for 52 genes. The observed lack of effects in the liver was ascribed to the upstream reduction and consequent detoxification of Cr(VI), firstly in the lung, then in the blood of the general circulation and finally in the liver itself.

## 6. Concluding Remarks

While the results obtained in the studies published thus far do not constitute a direct proof of a link between the stress response and Cr(VI)-induced carcinogenesis, they do show the ability of this carcinogen to modulate the expression of several components of this response under conditions of biological relevance. It has also become clear that the observed effects are dependent on tissue, cell type, Cr(VI) concentration, time of exposure and HSP isoform. Thus, future studies must address the issue of biological relevance and should also include adequate time courses, as it has been shown that HSP transcript and protein levels changed over time during the recovery period. Only through rational and solid experimental designs will it be possible to make further advances in this field and unequivocally determine whether the stress response does play a role in Cr(VI)-induced carcinogenesis.

## Figures and Tables

**Table 1:** Cr(VI)-induced effects on the expression and activity of components of the stress response.

Protein Family	System ^1^	Exposure Regime		Effect ^3^	Study
		Cr(VI) Dose/Concentration ^2^	Duration		
Small HSP	Rat lung epithelial cells	10 µM	24 h	Increased HSP10 protein levels	[42]
	HaCaT cells	7.4 µM	24 h	Increased HSP27 (HSPB1) mRNA and protein levels; Reduced HSP27 (HSPB1) phosphorylation	[43]
	Human primary skin fibroblasts	1 µM	16 h	Increased protein levels of phosphorylated HSP27	[44]
	BNL CL.2 cells	15 µM	3 h	Increased liver HSP27 protein levels	[45]
	ICR mice	10 mg/kg body weight (intraperitoneal injection)	8 weeks		
	BEAS-2B cells	10 µM	4 h	Unchanged HSP27 (HSPB1) mRNA levels	[46]
Hsp40	BEAS-2B cells	10 µM	4 h	Decreased HSP40 mRNA levels	[46]
HSP60	BEAS-2B cells		4 h	Decreased HSP60 (HSPD1) mRNA levels	
	Rat lung epithelial cells		24 h	Increased HSP60 protein levels	[42]
HSP70	BEAS-2B cells	1 µM	48 h	Unchanged Hsp72 (HSPA1A) protein levels; Decreased Hsp72 (HSPA1A) mRNA levels	[47]
		10 µM	4 h	Unchanged HSP70 (HSPA6), HSP70.1 (HSPA1) and HSP71 (HSPA8) mRNA levels	[46]
	Sprague-Dawley rats	0.25 mg/kg body weight (intratracheal instillation)	3 days	HSP70 mRNA levels increased in the lungs and unchanged in the liver; Unchanged HSP60, Grp75 and Grp94 mRNA levels in both lungs and liver	[48]
	HT29	10 or 50 µM	6 h	Increased Hsp72 (HSPA1A) mRNA levels	[49]
	HepG2 cells	0.5 or 1 µM			
		0.625–10 µM	48 h	Induction of HSP70 and Grp78 (HSPA5) promoters for [Cr(VI)] ≥ 5 µM	[50]
		100 µM	3 h	Unchanged HSP70 mRNA levels	[51]
	Primary culture of rat granulosa cells	10 µM	12 or 24 h	Decreased HSP70 protein levels	[52]
	L-02 cells	16 or 32 µM	24 h	Decreased HSP70 proteins levels	[53]
		8 or 16 µM	24 h	Increased Grp78 (HSPA5) mRNA levels	[54]
	BNL CL.2 cells	15 µM	3 h	Increased liver HSP70 protein levels	[45]
	ICR mice	10 mg/kg body weight (intraperitoneal injection)	8 weeks		
	A549	0.5 µM	2–24 h	Increased Grp78 (HSPA5) protein levels	[55]
HSP90	BEAS-2B cells	1 µM	48 h	Decreased Hsp90α (HSPC1) protein levels. Unchanged Hsp90α (HSPC1) mRNA levels	[47]
		10 µM	4 h	Decreased HSP90A (HSPC1) mRNA levels	[46]
	Primary culture of rat granulosa cells		12 or 24 h	Decreased HSP90 protein levels	[52]
	L-02 cells	16 or 32 µM	24 h	Decreased HSP90 protein levels	[53]
	A549	600 µM	2 h	Increased TRAP1 (HSPC5) mRNA levels	[56]
HSP100	Rat lung epithelial cells	10 µM	24 h	Increased HSP105 protein levels	[42]

^1^ A549, cell line established from a human lung adenocarcinoma; BEAS-2B, cell line established from human bronchial epithelium; BNL CL.2, cell line established from embryonic murine liver tissue; HaCaT, keratinocytes cell line established from human skin; HepG2, cell line established from a human hepatocellular carcinoma; HT29, cell line established from a human colorectal adenocarcinoma; L-02, cell line established from human embryonic liver tissue. ^2^ Cr(VI) was added as a K_2_Cr_2_O_7_ or Na_2_CrO_4_ aqueous solution. Of note, for several studies, Cr(VI) concentration values are ambiguous, as the expressions “*x* μM Cr(VI)” and “*x* μM potassium dichromate” were used indistinguishably, even though a given potassium dichromate concentrations corresponds to a Cr(VI) concentration twice that value. ^3^ For designations, see Section 3.1.

## Abbreviations

ATF	Activating transcription factor associated with stress
CAT	Chloramphenicol acetyl transferase
Cr	Chromium
Cr(III)	Chromium in the trivalent oxidation state
Cr(IV)	Chromium in the tetravalent oxidation state
Cr(V)	Chromium in the pentavalent oxidation state
Cr(VI)	Chromium in the hexavalent oxidation state
DSB	Double-strand break
ER	Endoplasmic reticulum
HSF1	Heat shock factor 1
Hsp	Heat shock protein (see Section 3.1)
HSP	Heat shock proteins (see Section 3.1)
MAM	Mitochondria-associated membrane
ROS	Reactive oxygen species
SSB	Single-strand break
TRAP1	Tumor necrosis factor receptor-associated protein 1
UPR	Unfolded protein response
UPRmt	Mitochondrial unfolded protein response
